# P-1425. Paxlovid Utilization and Social Vulnerability: Trends in Connecticut from 2022-2023

**DOI:** 10.1093/ofid/ofae631.1600

**Published:** 2025-01-29

**Authors:** Laura Hohenstein, Meghan Maloney, David Banach

**Affiliations:** University of Connecticut School of Medicine, West Hartford, Connecticut; Connecticut Department of Public Health, Hartford, Connecticut; UConn Health, Farmington, Connecticut

## Abstract

**Background:**

The COVID-19 burden disproportionately affects communities with lower socioeconomic status and high social vulnerability, as well as minority racial groups. Disparities have also been described in nirmatrelvir/ritonavir (Paxlovid) prescription rates across socioeconomic strata.

**Figure:**
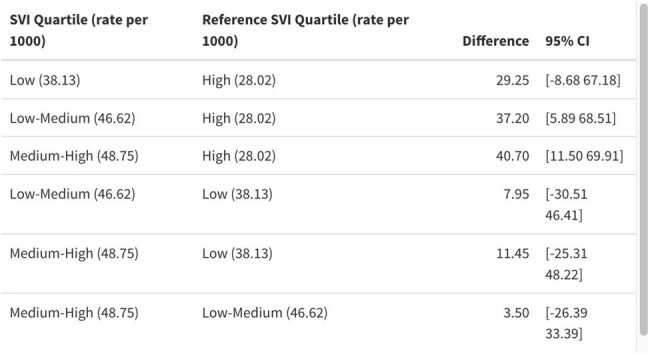

Differences in nirmatrelvir-ritonavir dispensing rates between SVI quartile groups from March 2022 – February 2023

**Methods:**

We performed an ecological analysis of associations between CDC social vulnerability index (SVI) and nirmatrelvir-ritonavir dispensing rates in Connecticut census tracts. SVI was categorized in four quartiles (low, low-medium, medium-high, and high). Nirmatrelvir/ritonavir dispensing locations were geocoded and dispensing rates per 1,000 population were calculated as the sum of courses dispensed per census tract during March 2022–February 2023. Independent-T tests were used to compare SVI in census tracts with and without medication dispensing sites. ANOVA with Tukey’s test was used to identify differences in dispensing rates by SVI quartile with paired t-test to identify significant differences before and after availability of the bivalent COVID-19 booster vaccine (March–August 2022 vs. September 2022–February 2023).

**Figure:**
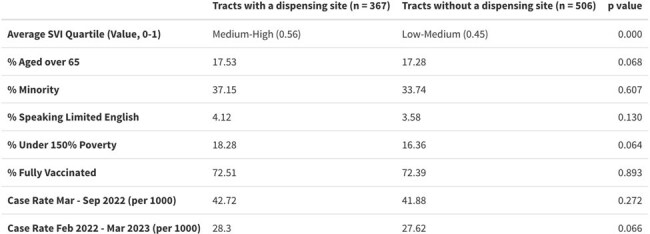

Demographic and COVID-19 characteristics of census tracts with and without a nirmatrelvir/ritonavir dispensing site

**Results:**

Mean SVI was higher in 367 census tracts with a dispensing site (mean 0.56) compared to 506 census tracts without a dispensing site (mean 0.45) (p < 0.01). Nirmatrelvir/ritonavir dispensing rates were lower in census tracts with high SVI (55.9 courses per 1000 people) compared to census tracts with low-medium SVI (93.1; difference -37.2 95% confidence interval (CI) [-68.5, -5.9]) and census tracts with medium-high SVI (96.6; difference -40.7, 95% CI [-69.9, -11.5]). Dispensing rates were higher after availability of the bivalent COVID-19 booster vaccine compared to before (p < 0.05), except in census tracts with high SVI where there was no statistically significant difference.

**Figure:**
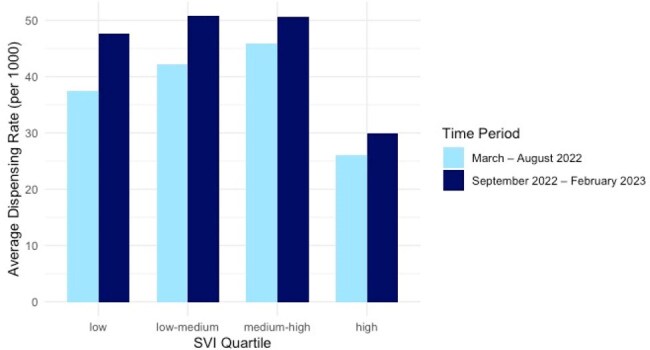

Nirmatrelvir/ritonavir dispensing rates per 1,000 population by census tract SVI quartile before and after availability of the bivalent COVID-19 booster vaccine

**Conclusion:**

Census tracts with high social vulnerability were less likely to have a dispensing site and had lower nirmatrelvir/ritonavir dispensing rates per 1,000 population. Observed differences in dispensing might signify inequities in healthcare access or medication prescribing. Findings highlight the importance of understanding and addressing barriers to accessing therapeutics in high vulnerability areas during public health emergencies.

**Disclosures:**

**Meghan Maloney, MPH**, Pfizer Global R&D: Former employee, separated in 2009. I do have a retirement entitlement which I do not actively manage/no stock options. PGRD offers periodic buy outs

